# Antiseptic after Treatment of Vaccination

**Published:** 1890-03-29

**Authors:** 


### ANTISEPTIC AFTER-TREATMENT OF VACCINATION.

Dr. Ballard and others have pointed out the difference between erysipelas from, and erysipelas after vaccination, the former resulting from the introduction of septic matter with the lymph, for which the vaccinator is to blame; and the latter, which is by far the more frequent, from the absorption of such matter after the vesicals have broken and exposed a receptive surface. A very reasonable suggestion for preventing this unfortunate occurrence is to cover the part from the eighth to the fourteenth day with a pad of boric or eucalyptus wool, or Hartmann's sublimated wood wool, and over this a layer of antiseptic gauze. Such an arrangement would not only effectually exclude the entrance of infective germs from without, but what parents will better appreciate, afford a protection against abrasion or premature separation of the scabs, in every way preferable to the dangerous and objectionable shields so much in vogue.

